# Phenotypic and Functional Characteristics of Exosomes Derived from Irradiated Mouse Organs and Their Role in the Mechanisms Driving Non-Targeted Effects

**DOI:** 10.3390/ijms21218389

**Published:** 2020-11-09

**Authors:** Seda Tuncay Cagatay, Ammar Mayah, Mariateresa Mancuso, Paola Giardullo, Simonetta Pazzaglia, Anna Saran, Amuthachelvi Daniel, Damien Traynor, Aidan D. Meade, Fiona Lyng, Soile Tapio, Munira Kadhim

**Affiliations:** 1 Department of Biological and Medical Sciences, Oxford Brookes University, Oxford OX3 0BP, UK; stuncay-cagatay@brookes.ac.uk (S.T.C.); amayah@brookes.ac.uk (A.M.); 2 Laboratory of Biomedical Technologies, Italian National Agency for New Technologies, Energy and Sustainable Economic Development (ENEA), 00123 Rome, Italy; mariateresa.mancuso@enea.it (M.M.); paola.giardullo@enea.it (P.G.); simonetta.pazzaglia@enea.it (S.P.); annasaran60@gmail.com (A.S.); 3 Department of Radiation Physics, Guglielmo Marconi University, 00193 Rome, Italy; 4 Centre for Radiation and Environmental Science, FOCAS Research Institute, Technological University Dublin, D08 NF82 Dublin, Ireland; amuthachelvi.daniel@dit.ie (A.D.); damien.traynor@tudublin.ie (D.T.); aidan.meade@tudublin.ie (A.D.M.); fiona.lyng@tudublin.ie (F.L.); 5 Institute of Radiation Biology, Helmholtz Zentrum München, 85764 Munich, Germany; soile.tapio@helmholtz-muenchen.de

**Keywords:** exosomes, ionising radiation, non-targeted effects, signalling

## Abstract

Molecular communication between irradiated and unirradiated neighbouring cells initiates radiation-induced bystander effects (RIBE) and out-of-field (abscopal) effects which are both an example of the non-targeted effects (NTE) of ionising radiation (IR). Exosomes are small membrane vesicles of endosomal origin and newly identified mediators of NTE. Although exosome-mediated changes are well documented in radiation therapy and oncology, there is a lack of knowledge regarding the role of exosomes derived from inside and outside the radiation field in the early and delayed induction of NTE following IR. Therefore, here we investigated the changes in exosome profile and the role of exosomes as possible molecular signalling mediators of radiation damage. Exosomes derived from organs of whole body irradiated (WBI) or partial body irradiated (PBI) mice after 24 h and 15 days post-irradiation were transferred to recipient mouse embryonic fibroblast (MEF) cells and changes in cellular viability, DNA damage and calcium, reactive oxygen species and nitric oxide signalling were evaluated compared to that of MEF cells treated with exosomes derived from unirradiated mice. Taken together, our results show that whole and partial-body irradiation increases the number of exosomes, instigating changes in exosome-treated MEF cells, depending on the source organ and time after exposure.

## 1. Introduction

Radiation affects not only targeted cells but also non-irradiated neighbouring cells, a response described as radiation-induced bystander effects (RIBE). Molecular communication between irradiated and unirradiated neighbouring cells initiates RIBE and out-of-field (abscopal) effects [1]. Abscopal effects are not always categorised as completely separate from RIBE, and both are examples of non-targeted effects of ionising radiation (NTE) [2]. NTE, particularly RIBE, represents a plethora of other biological effects such as DNA damage, epigenetic changes, changes in proliferation, and apoptosis observed in non-targeted cells and tissues that have received molecular signals produced by irradiated cells via intercellular communication through cell gap junctions or through soluble secreted factors [3,4,5,6,7,8]. A relatively new mechanism that has been identified for mediating RIBE by soluble secreted factors is intercellular communication via exosomes.

Exosomes, which are one type of cell-derived vesicle, exist in different biological conditions and serve as an important additional pathway for signal exchange between cells. They are small (30–120 nm of diameter) membrane vesicles of endosomal origin that are secreted by normal or pathological cells into the microenvironment [9,10,11,12]. Exosomes carry a variety of bioactive molecules including proteins, mRNA, microRNA, DNA, and lipids [13,14,15,16,17]. Exosomes can serve as mediators of cell–cell communication. Upon internalisation, they can release their bioactive cargo molecules, which can change molecular profile, signalling pathways, and gene regulation in the recipient cells [18]. Although functions of exosomes have been extensively studied in many fields, including neurodegenerative diseases [19,20,21] and cancer [22,23,24,25,26,27], their roles in radiobiology have not been recognised until recently. Exosomes secreted by irradiated cells are likely to engage in various aspects of the systemic response to ionising radiation (IR), including RIBE, as well as abscopal effects. Recent studies have shown that exosomes derived from irradiated cells can cause ionising radiation-induced effects in unirradiated recipient cells [28,29,30,31]. Moreover, studies have also shown that exosomes play a role in RIBE in cancer cells and resistance to radiotherapy [32,33,34,35,36]. Mechanistically, IR causes changes in exosomal secretion patterns and content of exosomal cargo of the target cells, which can cause RIBE in the bystander cells as shown in several in vitro studies. [30,32,37,38,39].

In vivo partial body irradiation (PBI) constitutes a major problem in radiation protection, with contradictory evidence suggesting that PBI may contribute to and/or protect against detrimental health effects. PBI exposures are the norm in diagnostic radiology, radiation therapy and occupational exposures and may have significant implications for systemic consequences and human health effects at low and intermediate doses of ionising radiation [40,41,42,43]. However, to date, only limited mechanistic studies are available in understanding the consequences of PBI effects.

Diagnostic X-rays are the primary human-made source of radiation exposure to the general population, accounting for 14% of the total annual exposure worldwide from all sources. Estimates of risk of cancer from these exposures are ranged from 0.6% to 3.0% based on the annual number of diagnostic X-rays undertaken in developed countries [44]. For a better understanding of the underlying processes, epidemiological evidence mounting this risk should be augmented with radiobiological justifications. Moreover, radiation is the mainstay of cancer therapy, as radiotherapy is used in more than 50% of localised patients and is an indispensable component of comprehensive cancer treatment and care [45,46]. Therefore, it is vital to elucidate key mechanisms that could increase the risk of secondary malignancies, which could be developed as a result of non-targeted effects.

The risks posed by partial body as well as whole body exposures after both low, and therapeutic doses for cancer and non-cancer endpoints can be evaluated for radiation protection purposes if there is a plausible and consistent mechanism for detriment, a dose–response relationship that allows risk assessment, and biomarkers of response available for molecular epidemiological analysis. Exosomes can be ideal candidates as both prognostic and predictive biomarkers to monitor the radiation response and risk assessment given that radiation affects not only the production of exosomes but also their composition.

Growing evidence has suggested that radiation therapy can result in an increase in the release of exosomes and increase in oncogenic materials within the exosomes. Studies with glioblastoma multiforme cell lines have shown that radiation can elevate exosome release with a molecular profile containing an abundance of molecules essential for cell motility such as connective tissue growth factor (CTGF) mRNA and insulin-like growth factor binding protein 2 (IGFBP2) protein [32]. Evidence also showed that head and neck squamous cell carcinoma (HNSCC) FaDu cells release exosomes with a different proteome profile compared to the unirradiated control cells [47]. Other studies carried out with irradiated HNSCC cells demonstrated that exosomes carry pro-survival signals following ionising radiation [34]. Exosome transfer from stromal to breast cancer cells can regulate therapy resistance pathways including the STAT1 pathway. It has been recently reported that in glioblastoma, exosomes increase the cancer cell’s ability to survive radiation by increasing oncogenic cargo and decreasing tumour-suppressive cargo [48].

Despite the extensive range of studies that have been carried out in exosome-mediated functions in radiation therapy and oncology, there is a lack of knowledge regarding the role of exosomes derived from inside and outside of the radiation field in the initial and delayed induction of non-targeted effects of ionising radiation. We have therefore investigated the role of exosomes as possible molecular signalling mediators of radiation induced out-of-target effects/RIBE. Exosomal characteristics were investigated by qNano analysis, TEM, Western blot and Raman spectroscopy. In vitro functional effects were investigated by transfering exosomes derived from whole body irradiated (WBI) or partial body irradiated (PBI) mice after 24 h and 15 days post-irradiation to recipient mouse embryonic fibroblast (MEF) cells and evaluating their effect on cellular viability, DNA damage and calcium, reactive oxygen species and nitric oxide signalling compared to that of exosomes derived from unirradiated mice. Taken together, our results show that whole and partial-body irradiation increases the number of exosomes, instigating changes in exosome-treated MEF cells, depending on the source organ and time after exposure.

## 2. Results

### 2.1. Characterisation of Exosomes

Exosomes were extracted from plasma at 24 h and from organ samples (brain, liver, and heart) at 24 h and 15 days post different IR exposure conditions by the ultracentrifugation method. Exosome concentration and their size distribution, presence of exosome markers and the biochemical profile of exosomes were evaluated by qNano analysis, TEM, Western blot and Raman spectroscopy. Significant differences were particularly observed in exosome number between samples, as fully described below.

#### 2.1.1. Characterisation of Exosomes by qNano

**Figure 1:**
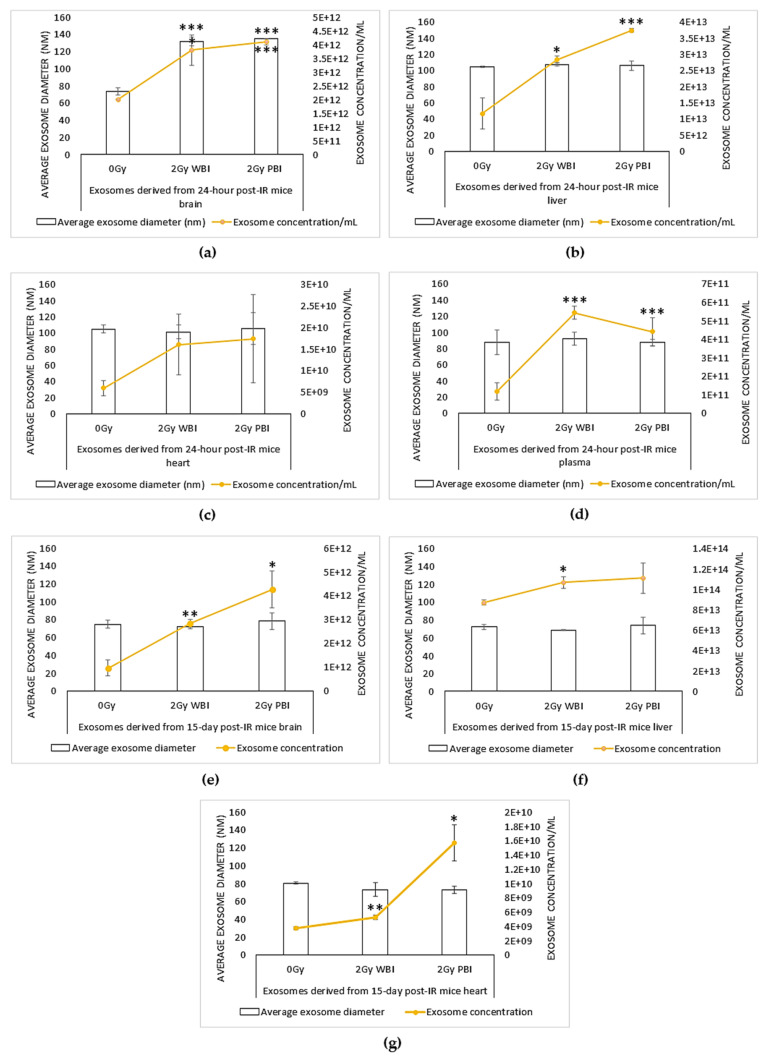
Concentration (exosome/mL) and size (nm) distribution of exosome suspensions obtained from (**a**–**d**) 24 h post ionising radiation (IR) and (**e**–**g**) 15 days post-IR 2 Gy whole body irradiated (WBI) and 2 Gy partial body irradiated (PBI) mouse compared to unirradiated mouse organs (brain, liver, heart) and plasma. Bars represent mean ± SD; significance was tested by Student’s *t*-test (* *p* < 0.05, ** *p* < 0.01, *** *p* < 0.001).

Exosome size and concentration of 2 Gy WBI and PBI mice organs (brain, liver, and heart) and plasma samples were assessed by using a tunable resistance pulse sensing (TRPS) system via qNano after 24 h and 15 days after irradiation (Figure 1).

Although exosome concentration levels varied for all organs and plasma, they were increased in the brain, liver, heart, and plasma of both 2 Gy X-ray irradiated WBI and PBI mice compared to the 0 Gy control groups after 24 h post-IR. Exosome concentrations in PBI mice organs showed a more dramatic increase compared to the WBI organs, while plasma obtained from WBI irradiated mice showed a higher level of exosomes compared to the plasma obtained from PBI mice.

For 15 days post-IR samples, exosome concentrations increased in the brain, liver, and heart of both 2 Gy WBI and PBI mice compared to the 0 Gy control groups. Taken together, the results show that ionising radiation can increase the yield of exosomes derived from 2 Gy mice organs after 24 h (Figure 1a–d) and 15 days (Figure 1e–g) following irradiation.

qNano analysis also showed that the exosome suspensions had a relatively narrow size distribution (70–130 nm) for all samples. Differences in the mean diameter of exosomes were significant for 2 Gy WBI and PBI brain samples compared to the unirradiated controls, whereas no significant difference in exosome size was observed between liver, heart, and plasma samples and their controls [30], indicating that ionising radiation can also alter the size distribution of exosomes in an organ-specific manner.

#### 2.1.2. Characterisation of Exosomes by Transmission Electron Microscopy (TEM)

**Figure 2:**
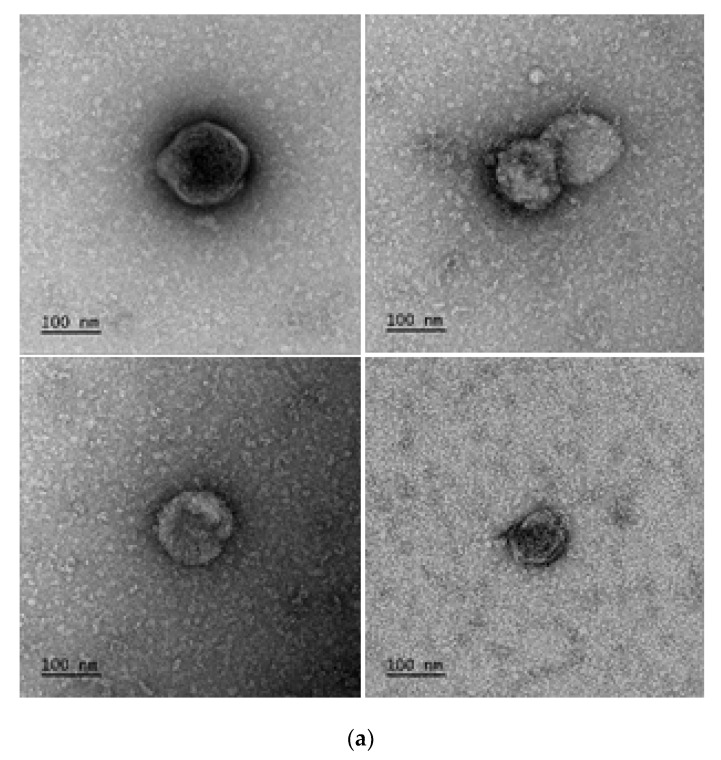

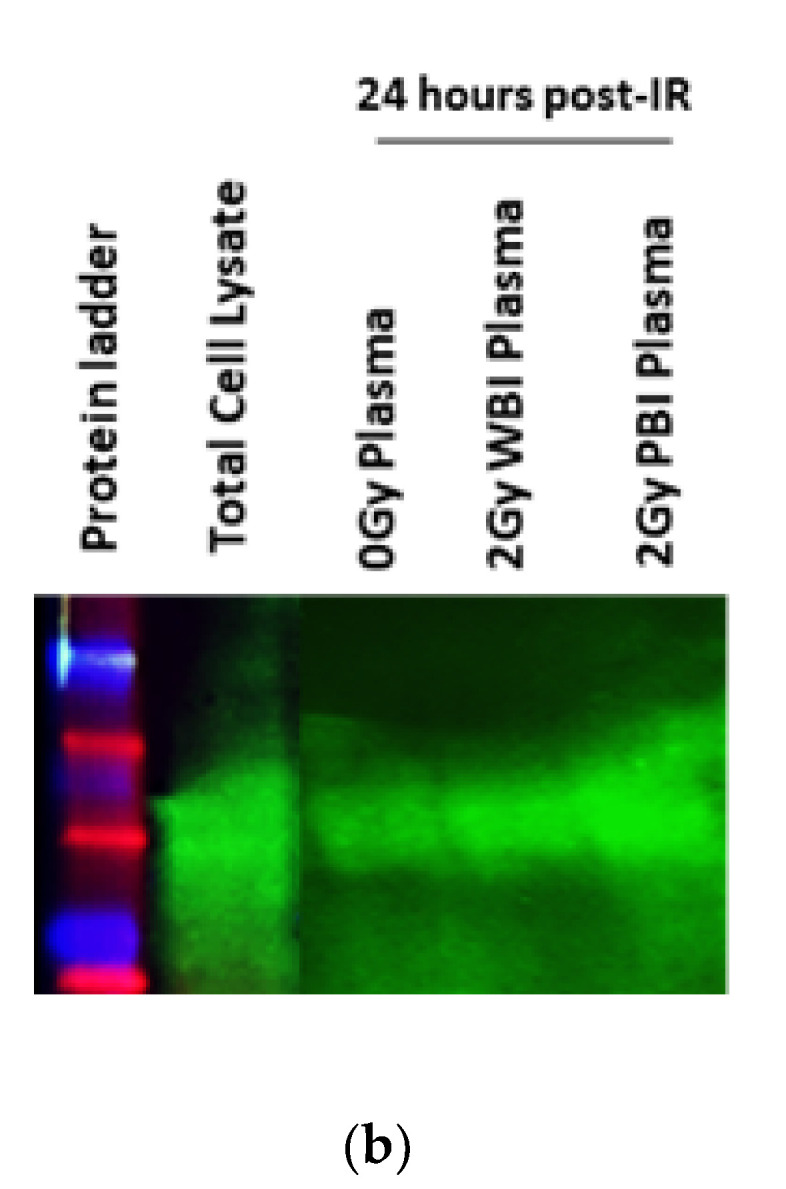
Confirmation of presence of exosomes. (**a**) TEM micrographs of exosomes. Representative images 1:10 or 1:100 diluted plasma exosome samples. (**b**) Western blot analysis of exosomes for CD63. Lane 1 protein ladder, lane 2: total cell lysate, lane 3: unirradiated (0 Gy) plasma sample, lane 4:2 Gy WBI plasma protein and lane 5:2 Gy PBI plasma protein sample.

Exosome suspensions were investigated by transmission electron microscopy for size. Negative staining of exosome suspensions showed vesicular structures in the anticipated size range and classical exosome morphology, as shown in Figure 2a.

#### 2.1.3. Characterisation of Exosomes by Western Blot

The presence of exosomes in extracted samples was confirmed by Western blot analysis against exosome marker CD63. A representative Western blot analysis for plasma exosome samples is shown in Figure 2b. CD63 bands of samples were observed in the predicted molecular weight (26 kDa), which further confirms the presence of exosomes together with findings from qNano analysis and electron microscopy.

#### 2.1.4. Characterisation of Exosomes by Raman Spectroscopy

**Figure 3:**
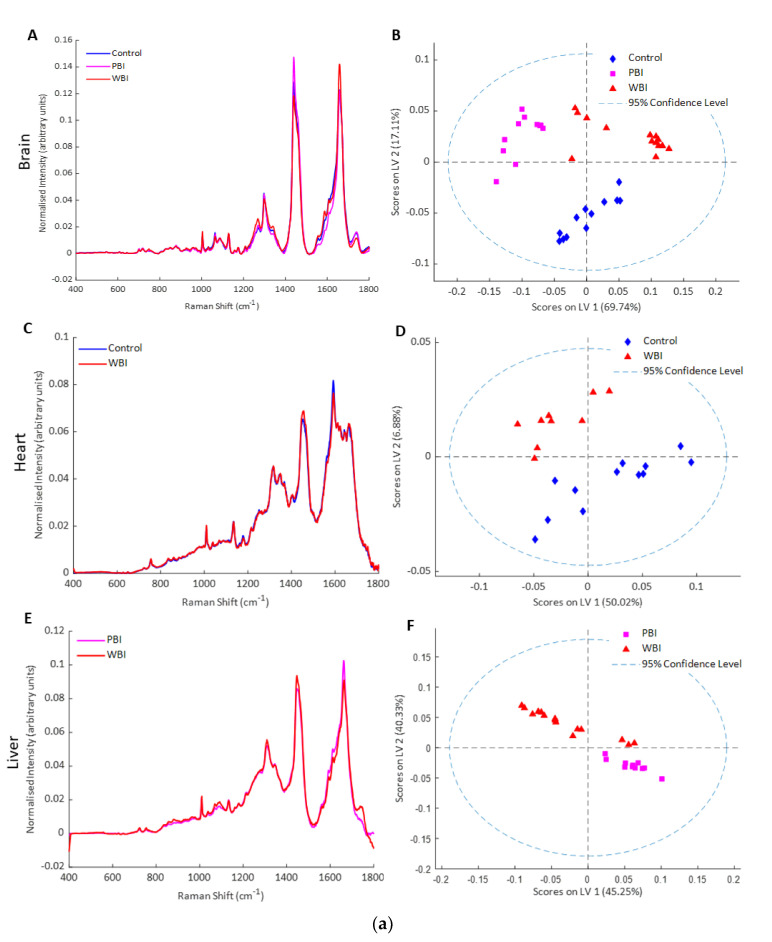

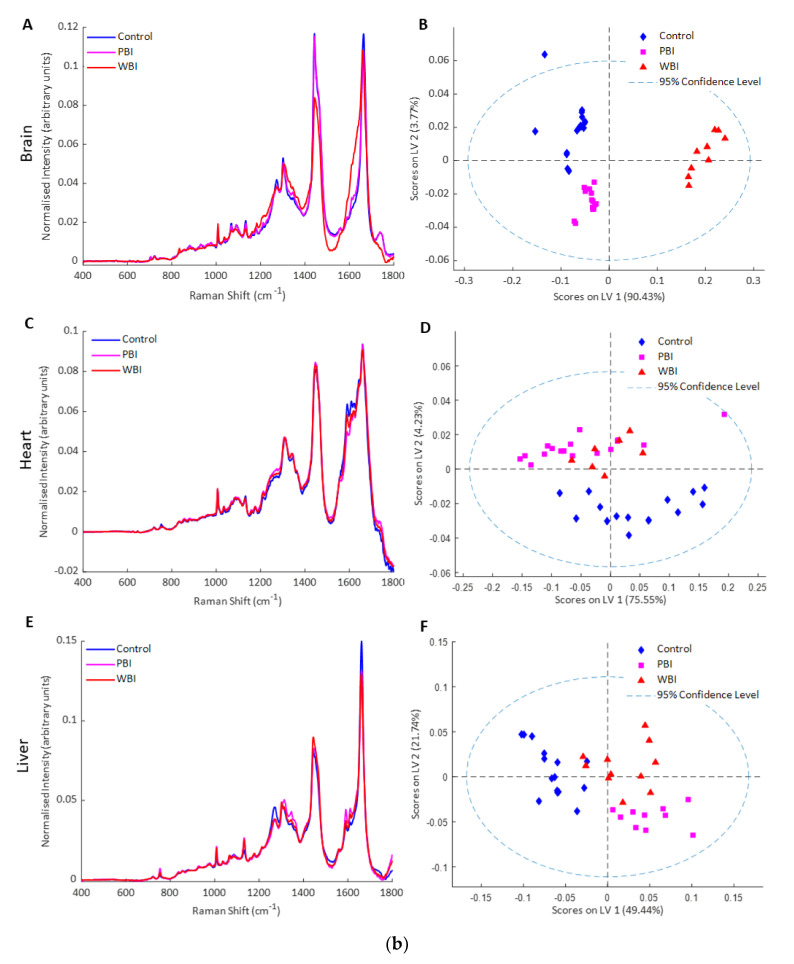
Raman spectroscopy of exosomes (**a**) 24 h post-IR: (**A**) mean Raman spectra from exosomes from brain of control, PBI and WBI mice at 24 h post irradiation, (**B**) PLSDA scatterplot of Raman spectral data from exosomes from control (blue), PBI (pink) and WBI (red) brain, (**C**) mean Raman spectra from exosomes from heart of control and WBI mice at 24 h post irradiation, (**D**) PLSDA scatterplot of Raman spectral data from exosomes from control (blue) and WBI (red) heart, (**E**) mean Raman spectra from exosomes from liver of PBI and WBI mice at 24 h post irradiation, (**F**) PLSDA scatterplot of Raman spectral data from exosomes from PBI (pink) and WBI (red) liver. (**b**) Fifteen days post-IR: (**A**) mean Raman spectra from exosomes from brain of control, PBI and WBI mice at 15 days post irradiation, (**B**) PLSDA scatterplot of Raman spectral data from exosomes from control (blue), PBI (pink) and WBI (red) brain, (**C**) mean Raman spectra from exosomes from heart of control and WBI mice at 15 days post irradiation, (**D**) PLSDA scatterplot of Raman spectral data from exosomes from control (blue) and WBI (red), (**E**) mean Raman spectra from exosomes from liver of control, PBI and WBI mice at 15 days post irradiation, (**F**) PLSDA scatterplot of Raman spectral data from exosomes from control (blue), PBI (pink) and WBI (red) liver.

Exosomes were also analysed by Raman spectroscopy, a label-free method based on light scattering which provides a biochemical profile of the sample [49,50]. Partial least squares discriminant analysis (PLSDA) showed good separation of the Raman spectral data from exosomes from brain, heart, and liver into control, PBI and WBI groups at 24 h and 15 days post-irradiation (Figure 3a,b). The spectral differences were based on changes in nucleic acid and protein features and these will be analysed in more detail in future work.

### 2.2. Effects of Exosomes from WBI and PBI Mice on Bystander MEF Cells

#### 2.2.1. Effects of Exosomes on Cell Viability

**Figure 4:**
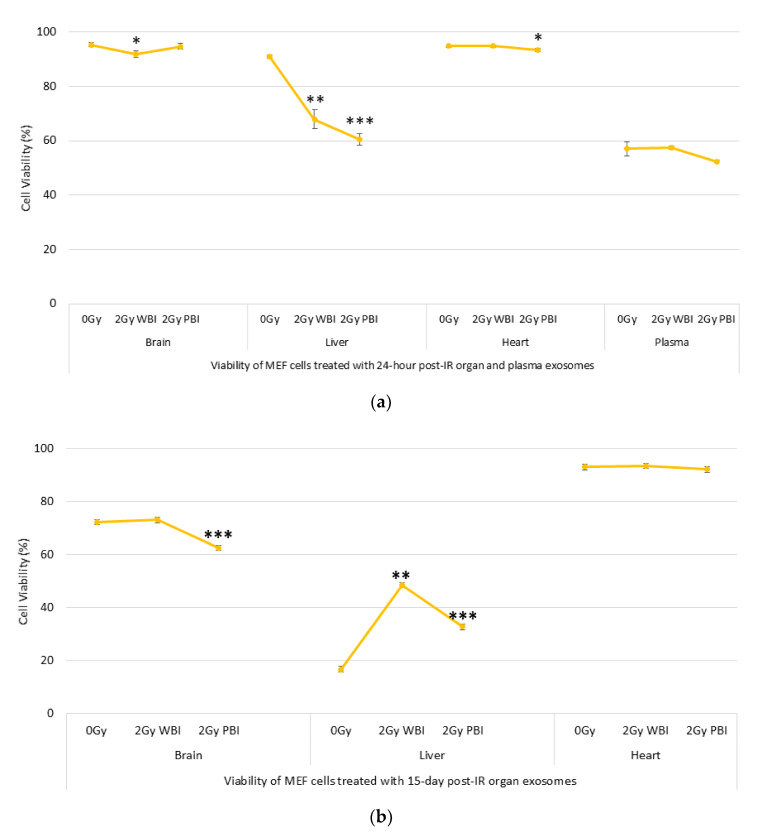
Viability of mouse embryonic fibroblast (MEF) cells treated with (**a**) 24 h post-IR and (**b**) 15 day post-IR exosomes obtained from organs and plasma of 2 Gy WBI or PBI mouse compared to unirradiated mouse organ and plasma exosomes. Data groups were obtained by triplícate measurements (* *p* < 0.05, ** *p* < 0.01, *** *p* < 0.001).

The effects of exosomes on cell viability were evaluated by using the MUSE Cell analyser. The results in Figure 4a show that 24 h post-IR, 2 Gy WBI brain, 2 Gy WBI liver, 2 Gy PBI liver and 2 Gy PBI heart exosomes significantly reduced the viability/survival of MEF cells. However, cells that received plasma exosomes did not show a significant change in the cell viability levels. High levels of cell death responses were observed in cells that received 15 days post-IR exosomes derived from organ samples, particularly the brain and liver, suggesting that exosomes can transfer long-lived signal-inducing radiation-induced genomic instability, as shown in Figure 4b. Moreover, a significant decrease in cell viability was also observed in cells treated with 15-day WBI and PBI exosomes compared to those that received 24-h WBI and PBI exosomes. Surprisingly, cells that received 0 Gy liver exosomes showed a high level of cell death compared to those that received WBI and PBI exosomes at the delayed time point (15-day exosomes), suggesting that delayed cell death can also be induced by exosomes derived from unirradiated liver cells. More investigations are needed to confirm this.

#### 2.2.2. Effects of Exosomes on DNA Damage

##### DNA Damage in Comet Tail

**Figure 5:**
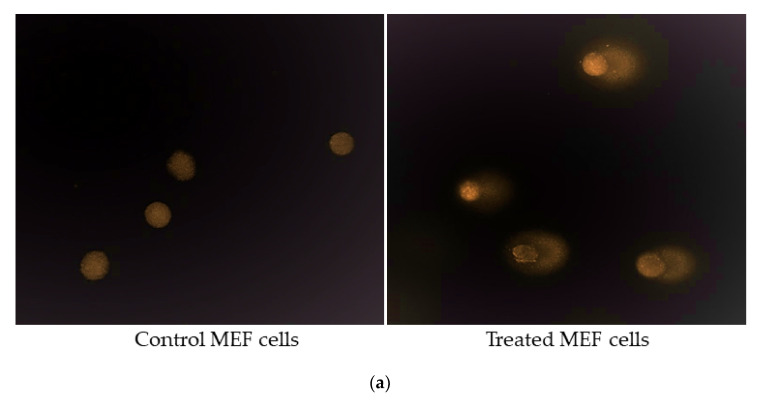

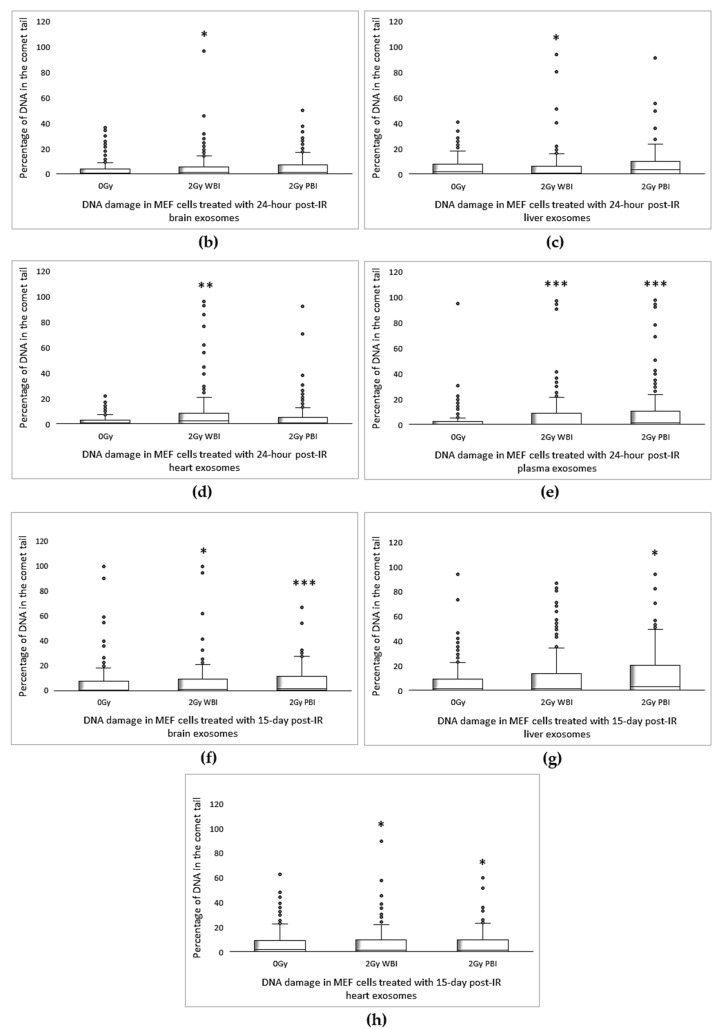
Comet assay showing the induction of DNA damage in MEF cells. (**a**) Representative fluorescent microscope images of untreated MEF cells and comet tails in treated MEF cells. (**b**–**e**) Induction of DNA damage in MEF cells treated with 24 h post-IR exosomes, (**f**–**h**) 15 day post-IR exosomes obtained from organs, and plasma of 2 Gy WBI or PBI mouse compared DNA damage in MEF cells treated with exosomes obtained from unirradiated mouse organ exosomes. Percentage of DNA in the comet tail was scored in 200 cells treated for each group. Statistical analysis was performed using the Mann–Whitney U Test (* *p* < 0.05, ** *p* < 0.01, *** *p* < 0.001).

Total DNA damage in the comet tail was measured in order to assess the role of exosomes obtained from 2 Gy WBI or PBI organs and plasma on the induction of DNA damage on the MEF recipient cells. As shown in Figure 5b–e, an increase in DNA damage was observed in 24 h post-IR brain, liver, heart, and plasma exosome-treated cells compared to unirradiated control groups. For brain and heart samples, significantly higher DNA damage was observed in MEF cells treated with 24 h 2 Gy WBI mice exosomes, while treatment with plasma exosomes from both 2 Gy WBI and PBI mice caused significantly higher DNA damage in MEF cells compared to unirradiated control treated MEF cells.

Similarly, as shown in Figure 5f–h, a significant increase in DNA damage was also observed in MEF cells treated with 15 days post-IR 2 Gy WBI and PBI brain, 2 Gy PBI liver, and 2 Gy WBI and PBI heart exosomes compared to their corresponding controls treated with unirradiated exosomes. In addition to the immediate bystander signal effects transferred by exosomes, exosome-transferred long-lived signals also have an ability to induce DNA damage responses in the treated cells.

##### γH2AX Immunostaining

**Figure 6:**
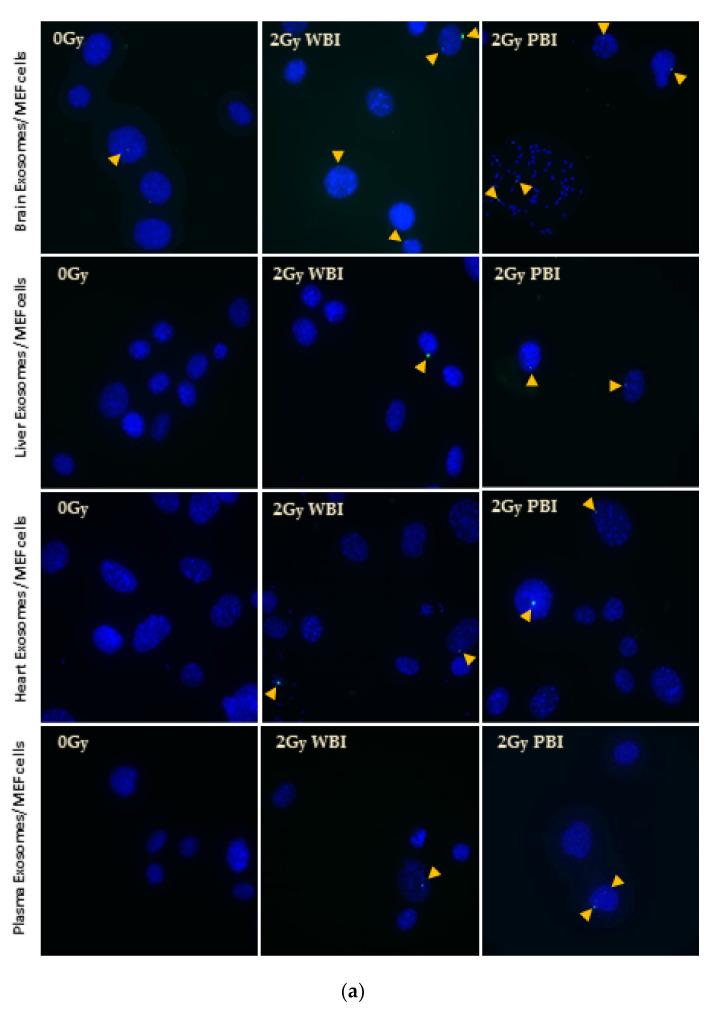

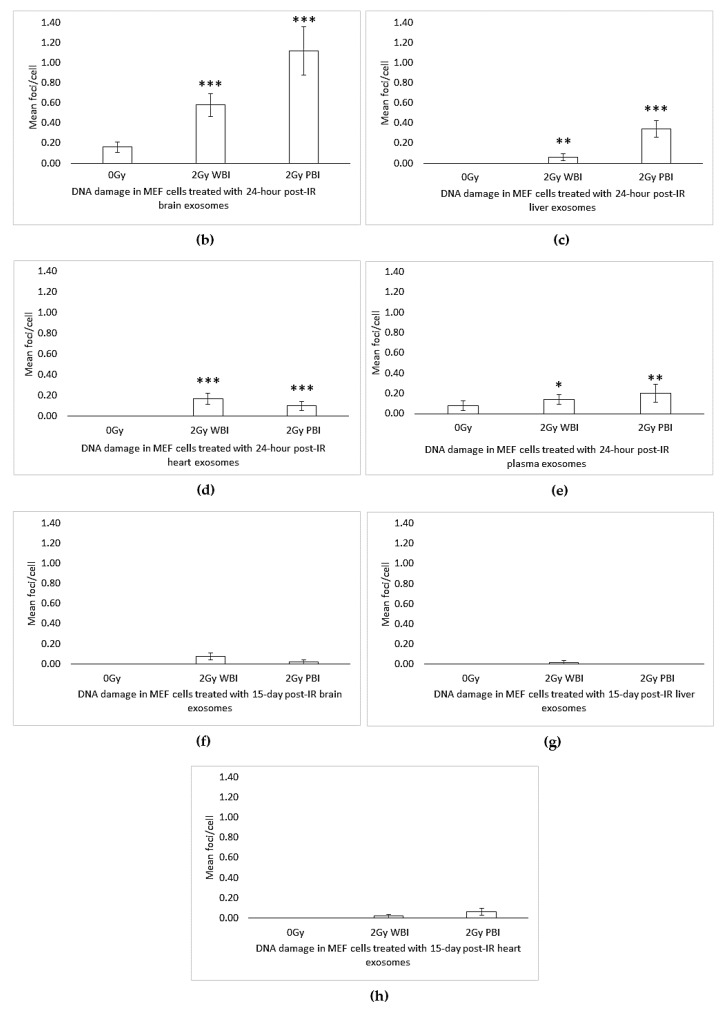
γH2AX foci formation in MEF cells after 24 h treatment with 24 h and 15 days post-IR organ and plasma of 2 Gy WBI or PBI mouse compared to unirradiated mouse organ exosomes treated cells. (**a**) Representative 63X fluorescent microscope images of MEF cells. Arrows indicate the location of γH2AX foci (Alexa488), cells were counterstained with 4′,6-Diamidine-2-phenylindole dihydrochloride (DAPI). (**b**–**e**) Bars represent the mean γH2AX foci formed per cell ± SEM after treatment with 24 h post-IR exosomes and (**f**–**h**) 15 day post-IR exosomes obtained from organs and plasma of 2 Gy WBI or PBI mouse compared to corresponding unirradiated mouse organ and plasma exosomes (* *p* < 0.05, ** *p* < 0.01, *** *p* < 0.001).

In order to further evaluate DNA damage in terms of Double-strand breaks (DSBs)γH2AX immunostaining was carried out in MEF cells treated with 2 Gy PBI or WBI mice organ and plasma exosomes and unirradiated organ exosomes for both 24 h and 15 day time points. DSBs were significantly higher in MEF cells treated with all 24 h post-IR organ exosomes and plasma exosomes, as shown in Figure 6b–e, while the highest levels of DSBs were observed in MEF cells treated with 24 h post-IR brain exosomes, as shown in Figure 6a,b. The foci count was not significant for 15 days post-IR exosome-treated MEF cells (Figure 6f–h), suggesting that 15 day post-IR exosome-induced DNA DSBs could be faithfully or unfaithfully repaired. Alternatively, severely DSB damaged cells may have been removed from culture, as significantly delayed cell death was observed in the treated cells, so the γH2AX method was unable to detect the breaks in the 15 days post-IR exosome-treated samples.

##### Chromosomal Aberrations

**Figure 7:**
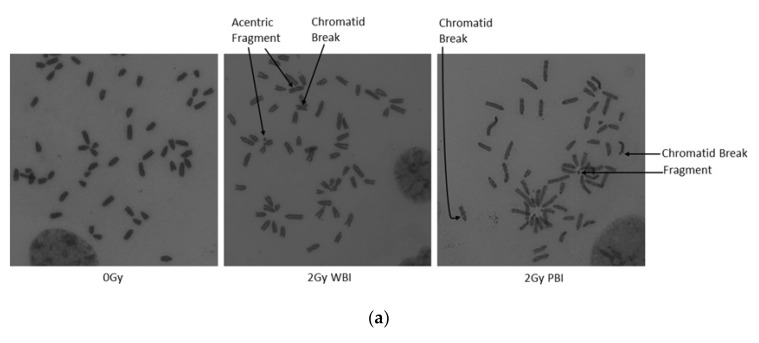

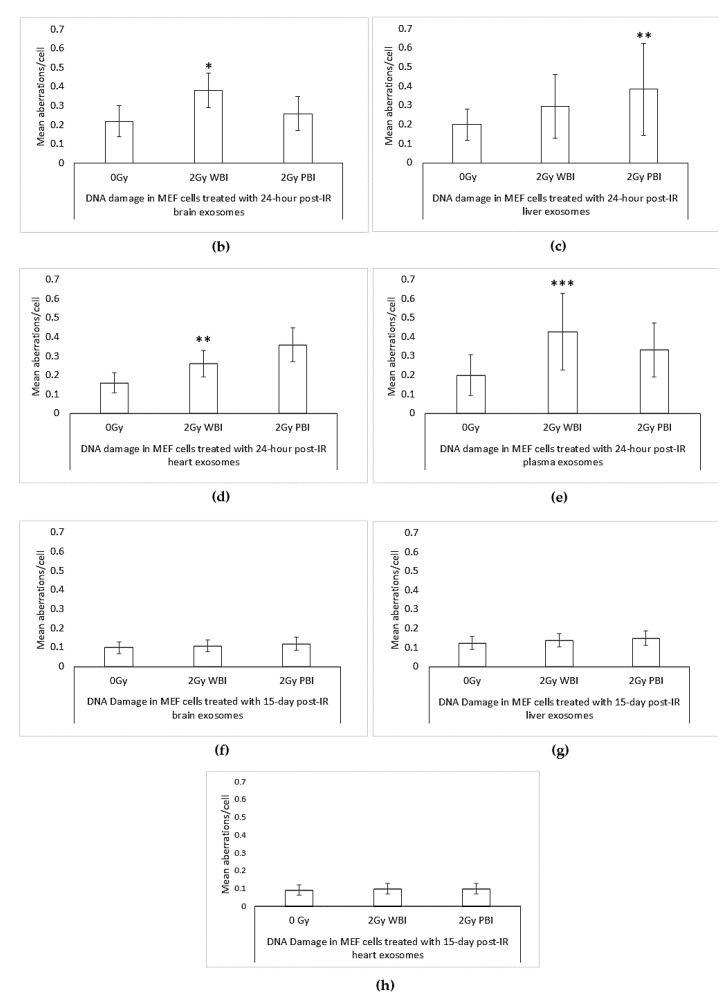
Chromosome analysis of MEF cells treated with 24 h and 15 days post-IR organ and plasma of 2 Gy WBI or PBI mouse compared to unirradiated mouse organ exosomes treated cells. (**a**) Normal and aberrant metaphase of MEF cells treated with exosomes derived from 24-h post-IR brain exosomes isolated from control (0 Gy), 2 Gy WBI and PBI mouse. Mean chromosomal aberrations/cell in MEF cells treated with (**b**–**e**) 24-h and (**f**–**h**) 15-day post-IR exosomes obtained from organs and plasma of 2 Gy WBI or PBI mouse compared to corresponding unirradiated mouse organ exosomes. Chromosomal aberrations (total) were scored in 100 metaphase spreads in cells treated with corresponding exosomes for 24 h. Bars represent mean chromosomal aberrations per cell ± SEM, significance was tested by Fisher’s exact test. (* *p* < 0.05, ** *p* < 0.01, *** *p* < 0.001).

Chromosomal damage was assessed in metaphase chromosomes of MEF cells treated with 24 h post-IR exosomes and 15 day 2 Gy WBI and PBI mice post-IR exosomes. As shown in Figure 7b–e, chromosomal aberrations were increased significantly in MEF cells treated with 24 h post-IR WBI brain, heart, and plasma and PBI liver exosomes compared to the MEF cells treated with control organs and plasma exosomes. However, chromosomal aberrations do not show significant differences between treatment groups for MEF cells treated with 15 days post-IR exosomes, as shown in Figure 7f–h. The findings suggest that exosomes derived from organs and plasma are able to induce chromosome aberrations in the MEF cells only at early time point, while the exosomes’ role in inducing chromosomal damage was reduced to the control level at the late time point post-exposure. This could be due to the ability of the cells to repair the damage at this time point of analysis.

#### 2.2.3. Role of Exosomes as Signalling Mediators

To further investigate the role of exosomes as mediators of radiation induced damage, calcium, reactive oxygen species (ROS), and nitric oxide (NO) were monitored in real time in MEF cells exposed to media containing exosomes from organs and plasma as well as from unirradiated, WBI, and PBI mice.

##### Calcium Signalling

**Figure 8:**
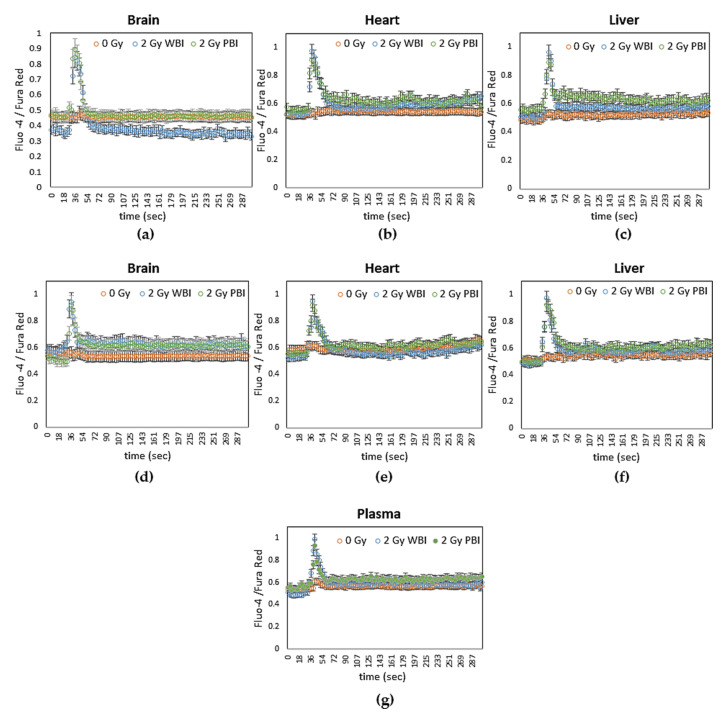
Intracellular calcium levels in MEF cells as indicated by the ratio of fluorescence emissions from the calcium sensitive dyes Fluo-4 and Fura Red after addition of media containing exosomes from (**a**–**c**) 24 h post-IR organs (**d**–**f**) 15 days post-IR organs and (**g**) 24 h post-IR plasma of 2 Gy WBI or PBI mice compared to corresponding unirradiated mouse organ and plasma exosomes.

Increases in intracellular calcium were measured using the calcium-sensitive dyes Fluo 3 and Fura Red. Rapid calcium fluxes were induced in MEF recipient cells following addition of exosomes from brain, heart and liver from WBI and PBI mice but not from sham irradiated mice at 24 h (Figure 8a–c) and 15 days (Figure 8d–f) post-irradiation. Similarly, rapid calcium fluxes were induced in MEF recipient cells following addition of exosomes from plasma from WBI and PBI mice but not from sham irradiated mice at 24 h post irradiation (Figure 8g).

##### ROS and NO Signaling

**Figure 9:**
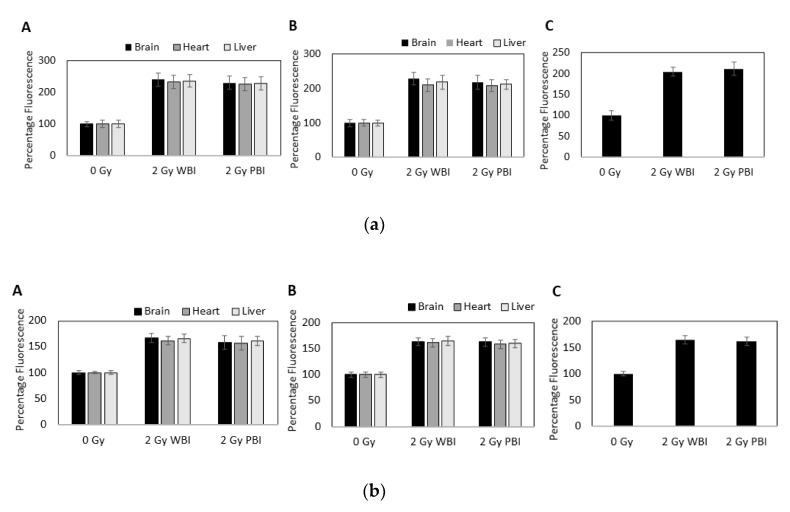
(**a**) Intracellular reactive oxygen species (ROS) levels in MEF cells as measured using CM-H2 DCFDA fluorescence after addition of media containing exosomes from brain, heart and liver from mice (**A**) 24 h and (**B**) 15 days after whole or partial body irradiation and (**C**) from plasma from mice 24 h after whole or partial body irradiation. Data are presented as mean ± SD after each sample was normalised to its respective control. (**b**) Intracellular NO levels in MEF cells as measured using DAF fluorescence after addition of media containing exosomes from brain, heart, and liver from mice (**A**) 24 h and (**B**) 15 days after whole or partial body irradiation and (**C**) from plasma from mice 24 h after whole or partial body irradiation. Data are presented as mean ± SD after each sample was normalised to its respective control.

Production of ROS in MEF cells was measured using a fluorescent dye, CM-H2 DCFDA. The data were expressed as mean fluorescence intensity normalised to each respective control. Significant ROS production was observed within 5 min of addition of exosomes from brain, heart and liver from WBI and PBI mice at 24 h and 15 days post irradiation and within 5 min of addition of exosomes from plasma from WBI and PBI mice at 24 h post irradiation as shown in Figure 9a.

Similarly, NO levels were measured using a fluorescent dye, 4-amino-5-methylamino-2,7-ifluorofluorescein diacetate (DAF-FM). Again, the data were expressed as mean fluorescence intensity normalised to each respective control. Significant NO production was observed within 5 min of addition of exosomes from brain, heart and liver from WBI and PBI mice at 24 h and 15 days post-irradiation and within 5 min of addition of exosomes from plasma from WBI and PBI mice at 24 h post-irradiation (Figure 9b).

## 3. Discussion

Exosome research is a new and growing field in radiobiology. To date, growing evidence supports the observation that ionising radiation can induce increased exosome release as well as changes in exosome content in in vitro models [32,34,51,52]. However, there is a lack of knowledge regarding the profile of exosomes released from directly irradiated versus abscopal organs and the systemic non-targeted effects of IR caused by those exosomes. Therefore, in the present study, we first investigated the organ responses to 2 Gy X-ray, a radiotherapeutic dose of ionising radiation, in terms of exosome profile including exosome concentration and size. Subsequently, we explored the role of these radiation-derived exosomes in the induction of cell death and DNA damaging effects in recipient MEF cells. Finally, the role of exosomes as mediators of radiation-induced damage was investigated by measuring calcium, ROS, and NO in exosome-exposed MEF cells.

Our data suggested that exosome yield can vary according to the organ type and whether exosomes were derived from directly irradiated organs (obtained from whole body irradiated animals) or abscopal organs (obtained from partial body irradiated animals). Differences in exosome concentrations between organs were also observed upon early (24 h post-IR) and delayed time (15 days post-IR) points.

Only significant change for size distribution of exosomes was observed in 24 h post-IR mice brain where WBI and PBI mice organs had an increased size distribution of exosomes compared to the unirradiated samples.

Although all three organs, the brain, liver, and heart, showed organ-specific exosome secretion levels, they exhibited a highly similar pattern of secretion when whole body and partial body irradiated sample organs were compared to their unirradiated counterparts at 24 h of post irradiation (Figure 1a–c). Both WBI and PBI organs showed a significantly increased level of exosome concentrations, particularly in the brain and liver, while exosome concentration levels were highest in PBI organs. One of the well-explained mechanisms of exosome release is linked to radiation-induced DNA damage and induction of the p53-related additional pathway of exosome biogenesis and secretion, which in turn leads to a significant increase in exosome release [52]. The same pattern of exosome secretion was also observed for all three organs at the delayed time point (Figure 1e–g).

Analysis of plasma samples from WBI and PBI mice, on the other hand, also showed significantly higher yield of exosomes for irradiated mice, albeit showing higher levels for WBI mice compared to PBI mice (Figure 1d). In addition, there was no alteration in size distribution between plasma samples.

Several studies support the involvement of exosomes in mediating RIBE in vivo and in vitro [29,30,37,53,54,55]. Bystander signals can be communicated through exosomes that can result in functional changes in target cells either by receptor-mediated interactions or by transfer of various bioactive molecules such as proteins, mRNA, miRNA or bioactive lipids carried by exosomes [56,57]. Therefore, in subsequent steps of our study, we interrogated whether WBI and PBI mice organ and plasma exosomes have an impact on the survival and DNA stability of recipient MEF cells.

It is evident from the literature that radiotherapeutic doses of X-ray can induce cell survival in cancer cell line models [34,48]. However, there has been a lack of information regarding impact of exosomes derived from both directly irradiated and abscopal organs on normal cells such as MEF cells, which was our model system in this study. Data showed that those exosomes from both early and delayed time points have an ability to reduce recipient MEF cell viability (Figure 4a,b). Albanese and co-authors showed that levels of TNFSF6 exfoliated on extracellular vesicles were increased following IR, suggesting a mechanism for abscopal and bystander effects after irradiation [58] which can also be one possible explanation for our findings.

Finally, we attempted to explore DNA damaging effects of exosomes in our model system as another aspect of bystander effects. Our findings show that both WBI and PBI organs and plasma induce DNA damage and chromosomal aberrations in the recipient MEF cells, where the extent of DNA damage was found to be organ and time specific. The amount of DNA in the comet tail showed significant results for both WBI and PBI organs when compared to MEF cells that received unirradiated organ and plasma exosomes as both early and delayed effects of IR, as depicted in Figure 5. Conversely, only early effects were significant when DSB foci (Figure 6b–e) or chromosomal aberrations (Figure 7b–e) were investigated. An absence of significant DNA DSBs or chromosomal aberrations in MEF cells that received delayed time point exosomes can be explained by the repair of DNA DSBs or removal of cells from the population (Figure 6f–h and Figure 7f–h) as a delayed time point response to IR. It has been shown that the phosphorylation status of critical DNA damage repair proteins can be changed by exosomes released from breast cancer cells [59]. Moreover, exosomes from irradiated HNSCC cells were shown to enhance DNA repair in unirradiated recipient cells [34].

In addition, our study showed the involvement of exosomes in mediating radiation damage by increasing calcium levels (within 30 s) and inducing ROS and NO (within 5 min) following addition of exosomes from brain, heart and liver from WBI and PBI mice at 24 h and 15 days post-irradiation and from plasma from WBI and PBI mice at 24 h post irradiation. Calcium, ROS, and NO signalling has been shown previously in bystander cells exposed to media from irradiated cells [60,61,62,63,64], and to exosomes from irradiated cells [29].

## 4. Materials and Methods

### 4.1. Animal Breeding, Irradiation and Sample Collection

Animal studies were performed according to the European Community Council Directive 2010/63/EU, approved by the local Ethical Committee for Animal Experiments of the ENEA on 01/10/2017 with the project identification code No.004/2017- SEPARATE, and authorized by the Italian Ministry of Health (n° 539/2018-PR). Eighty days of age C57 BL/6 female mice were either whole body irradiated (WBI) or partial body irradiated (PBI) with 2 Gy X-rays or sham irradiated (0 Gy).

For partial body exposure, the upper two-thirds of the adult mouse body was shielded, whilst exposing the lower one-third. At two different time points (24 h and 15 days) post-irradiation, animals were sacrificed by perfusion, washing out the blood and running physiological saline through the vascular system. The brains, livers and hearts were collected, snap frozen in liquid nitrogen, and stored at −80 °C for later exosome collection. Furthermore, at 24 h post-irradiation, animals were sacrificed, blood was collected and blood plasma were separated and snap frozen for later exosome extraction. All the samples were shipped to Oxford Brookes University (OBU) for exosome isolations as described below.

### 4.2. Exosome Isolation

The procedure of exosomes isolation from mouse organs (brain, liver and heart) were adapted from a protocol that has previously been established by Polanco et al. [65]. Briefly, organs (brain, liver and heart) were slowly defrosted, dissected and gently homogenised before being incubated in 7 mL of 20 units/mL papain (LS003119, Worthington, Lakewood, NJ, USA) in RPMI-1640 (R7388, Sigma, St Louis, MO, USA) for 20 min at 37 °C. Similarly, the reaction was stopped with 14 mL of ice-cold RPMI. The homogenised samples were then gently disrupted by pipetting with a 10 mL pipette, which was followed by a series of differential 4 °C centrifugations at 300× *g* for 10 min, 2000× *g* for 10 min, and 10,000× *g* for 30 min. However, the plasma samples were slowly defrosted, collected in falcon tubes and then similarly subjected to serial centrifugations at 300× *g* for 10 min, 2000× *g* for 10 min, and 10,000× *g* for 30 min. The supernatants from the 10,000× *g* centrifugations were passed through 0.45 μm and then 0.22 μm syringe filters, and then centrifuged at 120,000× *g* for 90 min at 4 °C to pellet exosomes. Exosome pellets were resuspended either in PBS or in exosome resuspension buffer (4478545, Invitrogen, Carlsbad, CA, USA) for downstream experiments.

### 4.3. Tunable Resistance Pulse Sensing (TRPS) via qNano

Exosome size and concentration were measured using TRPS via qNano machine (Izon Science™, Lyon, France), as described by Al-Mayah et al. [30], in which sample particles are driven through the nanopore (NP100 mm) by applying a combination of pressure and voltage.

Each particle causes a resistive pulse or blockade signal, which is detected and measured by the application software. Blockade magnitude is directly proportional to the volume of each particle [66], while blockade frequency is used to determine particle concentration [67]. Finally, magnitude and frequency values are converted to respective particle properties as size and concentration by normalizing against a known particle standard such as carboxylated polystyrene calibration nanoparticles.

### 4.4. Transmission Electron Microscopy (TEM): Morphological Analysis

In addition to qNano analysis and Western blotting to confirm existence of exosomes derived from organs and plasma, samples were morphologically analysed by electron microscopy [68]. In brief, 1:10 or 1:100 diluted exosome suspensions in PBS were incubated on formvar-coated and charged nickel grids (200 mesh) for 2 min. They were then fixed in 2.5% glutaraldehyde for 10 min, and then washed three times in 0.1 M phosphate buffer by dipping onto the surface of a water droplet and then stained with 8 μL of 2% aqueous uranyl acetate for 2 min. The stain was drawn off with cartridge paper to leave a thin negative stain. The grids were then examined and photographed under Jeol JEM-1400 Flash transmission electron microscope, with a Gatan OneView 16 Megapixel camera.

### 4.5. Western Blot

Western blotting has frequently been used for identifying and determining proteins, as one of the most commonly used methods in laboratories. It has also been utilised as a semi-quantitative technique in order to compare the expression of proteins in the cells and tissues [69].

Exosome proteins were extracted by using Total Exosome RNA and Protein Isolation kit (4478545, Invitrogen, Carlsbad, CA, USA). The protein content was measured using the modified Bradford assay using a Coomassie solution and Pre-Diluted Protein Assay Standards (23208, Thermo Scientific, Waltham, MA, USA). Exosome proteins were mixed with LDS sample buffer (NP0007, NuPAGE, Invitrogen, Carlsbad, CA, USA), and 30 μg from each sample were separated in a 4–20% polyacrylamide Mini-PROTEAN^®^TGX Stain-Free™ gels (456-8095, Bio-Rad, Hercules, CA, USA) and transferred onto Amersham^TM^ Hybond^TM^ PVDF membrane (10600090, GE Healthcare, Little Chalfont, UK). The membranes were blocked in 5% BSA and were incubated with the primary antibodies CD63 (ab 217345, Abcam, Cambridge, UK), in 5% BSA for 2 h at 1:1000 dilution. Membranes were washed three times with PBS-T which was followed by incubation with Goat Anti-Rabbit IgG H&L Alexa Fluor^®^ 488 (ab150077, Abcam, Cambridge, UK) at 1:10,000 dilution for 1 h.

### 4.6. Raman Spectroscopy

A LabRam HR confocal Raman instrument (HORIBA, Northampton, UK) was used for spectral acquisition. Manual calibration of the grating was carried out using the 520.7 cm^−1^ Raman line of crystalline silicon. Dark current measurement and recording of the substrate and optics signal was also performed, for data correction. As a source, a 532 nm laser of ~12 mW power was focused by a 100 X (MPlanN, Olympus, NA = 0.9) objective onto the sample; and the resultant Raman signals were detected using a spectrograph with a 1200 g/mm grating coupled with a CCD. Raman spectra were acquired in the 400 to 1800 cm^−1^ region. Multiple calibration spectra of 1,4-bis(2-methylstyryl) benzene were recorded along with each sample acquisition. All spectra were subsequently wavenumber calibrated using in-house developed procedures in Matlab v.9.3 (Mathworks Inc., Natick, MA, USA). The instrument response correction was performed using the spectrum of NIST Standard Reference Material (SRM) no.2242.

Raman spectroscopic data were pre-processed (normalization, baseline subtraction, etc.) using in-house developed protocols within the Matlab (The Mathworks Inc.) environment and corrected spectra were subjected to Partial Least Squares Discriminant Analysis (PLSDA).

### 4.7. Cell Culture and Exosome Treatments

Mouse embryonic fibroblast (C57 BL/6) (ATCC^®^SCRC-1008™) cells (MEF-BL/6-1) were grown in ATCC-formulated Dulbecco’s modified Eagle’s medium (30-2002, ATCC, Manassas, VA, USA) in the presence of 15% Fetal Bovine Serum (FBS) and 1% penicillin (P4333, Sigma, St Louis, MO, USA) and 5% CO_2_. A total of 1.5 × 10^6^ MEF cells were treated with exosomes obtained from tissue corresponding to the 1:5 of the organ mass in15 mL for 24 h prior to experiments.

### 4.8. Cell Count and Viability

Cell count and viability were measured by using Muse™ Count and Viability Cell Dispersal Reagent (MCH100107, Merck, Millipore, Kenilworth, NJ, USA) and Muse™ Cell Analyzer (0500-3115, Merck, Millipore, Kenilworth, NJ, USA) as described by Laka et al. [70]. Briefly, 380 μL Muse™ Count and Viability reagent was added to 20 μL cell suspensions in PBS, and incubated for 5 min. Then, cell viability was measured in Muse™ Cell Analyzer according to the dilution factor, 20. Viability percentages were evaluated. Each sample was analysed in triplicates.

### 4.9. Alkaline Single Cell Gel Electrophoresis (Comet Assay)

Single-cell gel electrophoresis, or the comet assay, is a sensitive method to quantify total DNA damage (double-strand breaks, single-strand breaks and base damage) in individual cells [71,72]. The comet assay was carried out as described by Al-Mayah et al. [28]. Briefly, microscope slides were coated with 1% normal melting point agarose (NMPA) (A9539, Sigma, St Louis, MO, USA), and were allowed to dry overnight. The coated slides were then placed on a metal tray on ice. Twenty thousand cells were resuspended with 200 μL of 0.6% low melting point agarose (LMPA) (BP165-25, Fisher Scientific, Pittsburgh, PA, USA) and placed immediately onto chilled pre-coated slides. The slides were then transferred to a Coplin jar, which was filled with cold alkaline lysis buffer (2.5 M NaCl, 100 mM EDTA pH 8.0, 10 mM Tris-HCl pH 7.6, and 1% Triton X-100, pH 10), and the jar was kept at 4 °C overnight.

The slides were then moved to a horizontal electrophoresis tank filled with electrophoresis buffer (0.3 M NaOH and 1 mM EDTA, (pH 13) at 4 °C for 40 min. The electrophoresis was run for 30 min, at 19 V, 300 A. Slides were neutralized with neutralizing buffer (0.4 M Tris-HCl, pH 7.5), washed with distilled water, and immediately stained with a 1:10,000 dilution of Diamond Nucleic Acid Dye (H1181, Promega, Madison, MA, USA). The slides were analysed using fluorescent microscopy and Comet Assay IV Image Analysis Software (Perceptive Instruments, Bury St Edmunds, UK). Tail intensities were evaluated for comparisons.

### 4.10. γH2AX Immunostaining

To investigate DNA damage in the exosome-treated cells, γH2AX Immunostaining assay was adapted from Zhang et al. [73]. Briefly, cells, fixed with 25% acetic acid in methanol, were dropped on slides and air-dried. Then cells were permeabilised with 0.2% Triton X-100 in PBS for 10 min. The cells were then blocked with 3% BSA in PBS for 1 h at room temperature (RT). Cells were incubated with γH2AX monoclonal antibody (ab26350, Abcam, Cambridge, UK) at 1:500 dilution overnight at 4 °C. Then, cells were washed three times with PBS-T and incubated with the secondary antibody conjugated with Alexa Fluor 488 (ab150113, Abcam, Cambridge, UK) for 1 h at RT at 1:1000 dilution. Cells were washed again three times with PBS-T and then mounted with anti-fade reagent containing DAPI.

### 4.11. Chromosome Analysis

Chromosomal preparation for Giemsa solid staining technique was carried out as described by [30]. Briefly, exosome-treated cells were incubated with 20 ng/mL demecolcine (D1925, Sigma, St Louis, MO, USA) for 1.5 h in a humidified 5% CO_2_ incubator at 37 °C. Cells were collected and centrifuged 300× *g* for 10 min at RT. Cell pellet was re-suspended with 0.075 M KCL as hypotonic solution for 20 min at 37 °C. The hypotonic cell suspensions were centrifuged at 200× *g* for 10 min at RT after addition of few drops of 25% acetic acid in methanol 3:1 fixative. Next, the cell pellet was fixed twice with 25% acetic acid in methanol. Fixed cells were dropped onto clean slides, and stained using the Giemsa solid staining technique. Slides were mounted and at least 100 metaphases were analysed per group.

### 4.12. Live Cell Imaging

Intracellular calcium levels were determined using Fluo 3 and Fura Red (Invitrogen/Molecular Probes, BioSciences, Dublin, Ireland) and ROS and NO were followed in real time using the fluorescent probes 5-(and 6-)chloromethyl 2,7 dichlorodihydrofluorescein diacetate, acetyl ester (CM-H2 DCFDA) (Invitrogen/Molecular Probes) and 4-amino-5-methylamino-2,7-ifluorofluorescein diacetate (DAF-FM) (Invitrogen/Molecular Probes) respectively, as previously described (Lyng et al., 2006).

Briefly, MEF recipient cells were grown on 35 mm glass bottom culture dishes (Mat Tek Corporation, Ashland, MA, USA; # P35 G-0-20-C). Twenty-four hours after plating, cells were washed twice with a buffer containing 130 mM NaCl, 5 mM KCl, 1 mM Na_2_ HPO_4_, 1 mM CaCl_2_ and 1 mM MgCl_2_ (pH 7.4) and incubated with 3 μM Fluo 3 and 3 μM Fura Red acetoxymethyl esters for 1 h and with 5 μM CM-H2 DCFDA or DAF-FM for 30 min in the buffer at 37 °C. Subsequently, the cultures were washed three times with buffer. All dyes were excited at 488 nm and fluorescence emissions at 525 (Fluo 3, CM-H2 DCFDA and DAF-FM) and 660 nm (Fura Red) were recorded using a Zeiss LSM 510 confocal microscope. Exosomes were added after 60 s when a stable baseline had been established. All measurements were performed at room temperature.

### 4.13. Statistical Analysis

For exosome size, diameter and viability data, significance was tested by Student’s *t*-test using raw data. Each experiment was carried out in triplicate. Analysis showed no significant inter-experimental variation; therefore, data from these experiments were pooled. For analysis of comet assays, statistical analysis was performed using the Mann–Whitney test, utilising the median of the raw data. Meanwhile, chromosomal analysis and γH2AX immunostaining data were subjected to Fisher’s exact test. Data were considered statistically significant if *p*-value was lower than 0.05 (* *p* < 0.05, ** *p* < 0.01, *** *p* < 0.001).

## 5. Conclusions

In this study, we provide the first insights into the in vivo systemic effects of early and delayed effects of X-ray irradiation in terms of exosome profile and bystander effects. Taken together, the findings show that exosome yield is organ-specific and can be significantly increased in both directly irradiated and abscopal organs. On the other hand, changes in the levels of survival and DNA damage in MEF cells, receiving PBI or WBI exosomes, were not necessarily correlated with the increase in exosome yield in different radiation conditions or time points. Those manifested bystander effects in MEF cells also differed as early and delayed responses to ionizing radiation. The role of exosomes in mediating radiation damage was shown by rapid calcium fluxes and induction of ROS and NO in MEF recipient cells following addition of exosomes from WBI and PBI mice, but not from unirradiated mice. Altogether, these results draw attention to the content of those exosomes, which can be the key to understand the observed effects. Further studies such as miRNA expression profiles and proteomics will help to discover the molecules that are responsible for the observation of bystander effects in recipient cells.

## Abbreviations

NTE	Non-targeted effects
RIBE	Radiation induced bystander effects
IR	Ionizing radiation
MEF	Mouse Embryonic Fibroblast
DSB	Doublestrand break
WBI	Whole body irradiation/irradiated
PBI	Partial body irradiation/irradiated
